# Recent Advances in Microfluidic Biofuel Cells

**DOI:** 10.3390/bios15090627

**Published:** 2025-09-20

**Authors:** Takahiro Kawaguchi, Shota Ito, Daisuke Nakane, Takashiro Akitsu

**Affiliations:** Department of Chemistry, Faculty of Science, Tokyo University of Science, 1-3 Kagurazaka, Shinjuku-ku, Tokyo 162-8601, Japan

**Keywords:** (bio)fuel cells, microfluidic fuel cells, enzyme immobilization, electron transfer, mediators

## Abstract

Traditionally, fuel cells operate by using small fuel molecules such as hydrogen and methanol to produce energy, water, and carbon dioxide. Enzyme biofuel cells use enzymes rather than precious metals as electrode catalysts. In recent years, enzyme-immobilized electrodes have been developed by combining enzyme biofuel cells with microfluidic technology to improve the efficiency and performance of fuel cells. In this review, we will provide an overview and describe the current status of recent enzyme biofuel cells, microfluidic technology, and their applications to microfluidic fuel cells.

## 1. Introduction

Traditionally, fuel cells use small molecules (e.g., hydrogen or methanol) to produce energy, water, and carbon dioxide (e.g., methanol oxidation). Fuel cells are typically catalyzed by precious metals such as platinum. The conversion of chemical energy to electrical energy can be achieved using a variety of fuels, such as methanol, glycerol, glucose, and lactic acid, and a variety of electrodes, including abiotic, biotic (enzymatic and microbial), and hybrid [1]. Fuel cells have a wide range of applications, including transportation, consumer electronics, and stationary power systems, As such, fuel cells are also considered a core technology element in the impending transition to renewable energy, and in the future, they hold promise for microfluidics to improve electrochemical energy conversion [2,3].

A biofuel cell is a type of fuel cell that uses a biological catalyst (enzyme or microorganism) instead of the typical abiotic catalyst. Enzymes have distinct advantages over chemical catalysts, including biocompatibility, high efficiency, high activity under mild conditions, and high selectivity [4]. Therefore, enzyme-based biofuel cells are also used.

The enzymatic catalysis of the fuel oxidation or oxidant reduction reactions between the enzyme and the electrode surface requires rapid and efficient electron transfer to the electrode surface [4]. To accelerate the electron transfer rate and increase the efficiency of fuel cells, electron mediators (cofactors) are frequently used [5]. The reduction potential of the mediator is important because it determines the normal operating potential of the electrode based on direct electron transfer. In order to elucidate the mechanism of electron transfer enzymes and improve the output of biofuel cells, it is necessary to design microfluidic fuel cells, develop efficient electrodes in terms of the durability and charge reduction in precious metals, and improve the performance of electrodes in (bio)fuel cells [1,4].

To overcome the challenges of stability and immobilization, a diversity of microfluidics designs and practical applications are considered. A micro fuel cell is defined as a device that operates on a virtual membrane created by separating the fuel and oxidant streams.

Fuel cells are attractive candidates for microelectromechanical systems (MEMS) applications because of their relatively simple structure and lack of moving parts, which allow for high yields in mass production. Furthermore, the growing demand for compact power sources for portable electronics and embedded devices in recent years has spurred interest in miniature fuel cells, which can achieve higher energy density than conventional batteries. Therefore, more surface area can be obtained for a given volume in microfluidic channels. Microfluidic microbial biofuel cells offer advantages over traditional cell structures, including faster mass transfer rates, higher surface-to-volume ratios, and faster response times.

For these reasons, it is expected that the combination of enzymes with microfluidic technology will bring special advantages to microbial biofuel cells. Also, unlike microorganisms and living organisms, enzymes react well with fuels and oxidants, eliminating [5]. There is active research into designing microfluidic fuel cells and improving their performance by developing efficient electrodes with excellent durability and charge reduction in precious metals.

In this review, taking into account the current situation and challenges described above, we will provide an overview of (enzymatic and microbial) biofuel cells and introduce recent progress in microfluidic biofuel cells.

## 2. Microfluidics for (Bio)fuel Cells

### 2.1. Applications and Examples of Microfluidics for Biofuel Cells

Microbial fuel cells can be classified into macro, meso, and micro sizes. Microfluidic microbial fuel cells are defined as small microbial fuel cells with a volume between 1 and 200 μL. Microfluidic (bio)fuel cells can also operate using membranes [6]. Electrodes, membranes, and fluid delivery systems are integrated into the microfluidic interior space and biochip. The small size of microfluidic microbial fuel cells makes them compatible with microfabrication techniques. Their miniaturization also offers significant advantages over microfluidic fuel cells (MFCs) of other sizes, including a high surface area-to-volume ratio (SAV), rapid response to reactants, and precise operation and performance. Table 1 above compares the performance and lifetime (power density, maximum cell voltage, oxidant, and output density) of fuel cell types.

On the other hand, the disadvantage of microfluidic microbial fuel cells, which means high operating costs. Alternatively, the immobilization of biocatalyst materials, microorganisms, and enzymes is a challenge for microfluidic applications [5]. The overall lack of power output is thought to be due to high internal resistance. A particular issue at the microscale is that as the size of the microfluidic cell decreases, viscous effects and surface forces become greater in microfluidic flows, while inertial and viscous forces become smaller [7].

**Figure 1:**
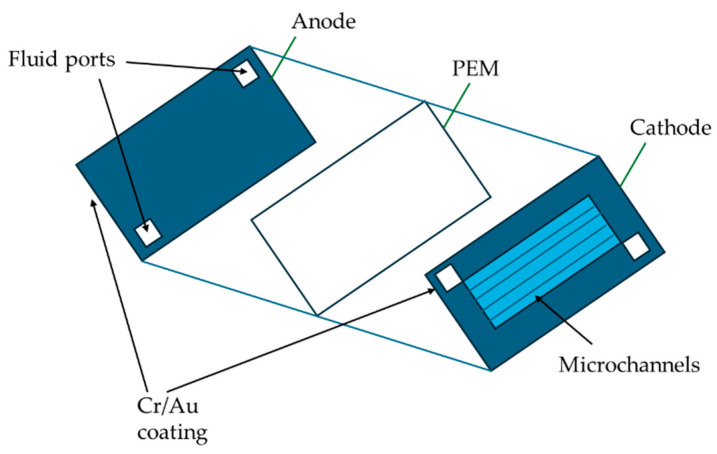
A microbial biofuel cell based on a bulk silicon micromachine. Adapted from Ref. [6].

As a practical application example, in this context, Chiao et al. [8] presented a bulk silicon micromachined microbial biofuel cell (Figure 1). A bulk silicon micromachined microbial biofuel cell shown in Figure 1, which is constructed in a sandwich structure with a (polymer electrolyte membrane) PEM sandwiched between Cr/Au-coated anode and cathode layers.

Moreover, Siu and Chao then used the same yeast *S. cerevisiae* to develop a soft lithography-based polydimethylsiloxane (PDMS) biofuel cell that converts the chemical energy stored in blood glucose in the bloodstream into electricity (namely, anodic reaction) [5]. Methylene blue was used as an “electron transfer agent” (i.e., moving mediator) to navigate the cell wall and reach the interior of the microorganism. More than 70,000 micropillars (8 μm, active electrode area up to 40 μm × 40 μm) on PDMS have significantly expanded the possibility of long-term miniature power sources for implantable devices by using human blood components as both electrolyte and fuel source with the help of microfluidic technology [5,9]. Furthermore, they presented a microfluidic biofuel cell based on the marine bacterium *S. oneidensis* MR-I strain and fueled by lactate. The vertically stacked 1.5 μL.

On a different note, in addition to the advantages in cell performance, enzymatic biofuel cells, when combined with microfluidic technology, offer special advantages over microbial biofuel cells as mentioned above. Unlike microorganisms and living organisms, enzymes react favorably with the fuel at the anode and the oxidant at the cathode, thus reducing the fuel crossover problem. It has been experimentally shown that oxygen dissolved in an aerobic solution containing biofuels such as glucose acts as a substrate for the enzyme reaction at the cathode and also as an inhibitor for the anodic reaction. Thus, the dissolved oxygen blocks electrons from the anode (enzyme and/or mediator), reducing the externally supplied power. This eliminates the need for a PEM, simplifies the structure, and expands the scope of application of microfabrication. In fact, such a typical cell consists of an oxygen cathode with adsorbed bilirubin oxidase (BOD) or an oxygen cathode with adsorbed glucose dehydrogenase (GDH), diaphorase, and vitamin K_3_. The enzyme biofuel cell consists of a glucose anode fabricated with co-immobilized_3_-pendant poly-*L*-lysine.

Here is an example of a material that acts both as an enzyme immobilizer and an electron transfer mediator for enzyme and electrode. The immobilization of enzymes on conductive supports for microfluidic applications was pioneered by Moore, Togo, and Heller et al., who reported that the power density of a biofuel cell in oxygen-saturated solution was 30% lower than in air due to undesired electron transfer from glucose oxidase (GOD) to dissolved oxygen. Therefore, it is necessary to combine the enzyme with VK_3_ (VK studied a glucose anode prepared by immobilizing alcohol dehydrogenase (ADH) as an electron mediator in a 100 μm deep microchannel). In combination with a supporting nicotinamide adenine dinucleotide (NAD). NAD methylene green immobilized electron transport layer, the microfluidic bioanode generated a current density of up to 53 μA cm^−2^.

**Figure 2:**
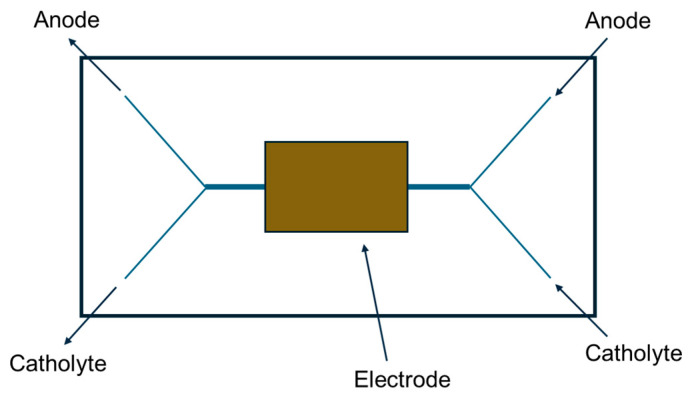
Overview of an enzymatic glucose/O_2_ microfluidic biofuel cell.

In the microscale environment, the absence of convective mixing promotes laminar flow of fluids, and fuel and oxidant streams can flow in parallel through the microchannels without the need for a membrane to prevent mixing. Protons diffuse across the liquid [10]. Togo et al. developed an enzyme-based bioanode that uses vitamins as mediators in the catalytic oxidation of NADH by K_3_Dp. Glucose is oxidized by a Dp/GDH enzyme bilayer, and the inner Dp layer is VK_3_-modified poly-*L*-lysine. They also developed an to allow efficient exchange of fuel and oxidant. It is reported that when supplied with a mixture of O_2_ (air-saturated glucose solution), the upstream cathode protects the downstream anode from interference by O_2_ molecules, resulting in improved cell performance [5]. This fuel cell, which uses an O_2_ Y-shaped microfluidic fuel cell structure developed by Zedba et al. [11], boasts the highest power density. The O_2_ Y-shaped microfluidic channel shown in Figure 2 has a tube diameter of 2 mm, and the central rectangle represents the electrode.

### 2.2. Selected Elemental Technology

#### 2.2.1. Materials for Microfluidic Design

Materials included a JB Weld epoxy, 100 mm silicon wafers, and Sylgard 184 (purchased from Ellsworth Adhesives, Germantown, WI), and isopropanol [11].

To create a dual-chamber micro-MFC, the anode chip was cleaned with 70% ethanol and then dried with nitrogen. The PEM, carbon cloth cathode, and PDMS stamp were carefully aligned with the tubing holes in the flow channels and laminated together, sandwiching the device between two acrylic plates. Thin carbon cloth wires and electrical wires were used to extend the anode and cathode, respectively, to the copper pins on the chip carrier, completing the circuit with an external load connected to the pins. Four sterile PE tubes were then inserted into two different holes on the PDMS stamp to create a path for liquid transport. The micro-MFC device assembled in the previous experiment had a 15 mm × 1 mm × 100 µm anode chamber and a 10 mm × 4 mm × 100 µm cathode chamber. Sterile water was made to flow through each chamber, demonstrating no leakage between the channels [12].

The PDMS channels were fabricated by applying SU-8 50 negative photoresist to a 4-inch silicon wafer at 1750 rpm for 30 s. The micromolding channels were fabricated using a similar method to the PDMS flow channel fabrication process, using a spin program of 1800 rpm for 30 s and SU-8 10 negative photoresist. The photoresist was prebaked at 90 °C for 5 min using near-UV flood nucleation, followed by UV exposure through a negative film containing the micromolded channels and flow channel design structures. After exposure, the silicon wafer was postbaked at 90 °C for 5 min. It was then developed with Nano SU-8 developer and the thickness of the photoresist was measured. This thickness corresponded to the channel depth of the PDMS structures to be fabricated later. Next, a 10:1 mixture of Sylgard 184 elastomer and curing agent was coated on the silicon wafer and cured at 75 °C for approximately 2 h. The resulting PDMS channels were 130 µm wide, 200 µm deep, 100 µm deep, and 3.0 cm long. The channels used for the electrode microchannels were 45 µm wide, 42 µm deep, and 2.5 cm long [11].

#### 2.2.2. Immobilization of Biocatalysts

From here on, we will focus on some of the elemental technologies. The technique of immobilizing biocatalysts on electrode surfaces offers many advantages when working with biomaterials. Immobilized biomaterials also usually have better adaptability to temperature changes and vibrations, which improves pH and stability and lifespan. This is advantage of biocatalyst immobilization.

A novel, versatile and effective method for immobilizing enzymes, called crosslinked enzyme aggregates (CLEAs), involves precipitating the enzyme from an aqueous buffer and crosslinking the resulting physical aggregates of enzyme molecules, allowing for rapid optimization. This method has been shown to be applicable to a wide range of enzymes, including various hydrolases as well as lyases such as nitrile hydratases and oxynitrilases, and oxidoreductases such as laccases and galactose oxidases. CLEAs are stable and recyclable catalysts that exhibit high catalytic productivity [13].

#### 2.2.3. Biochemical or Biomedical Applications of Microfluidic Devices

Chemical, biological, and medical applications of microfluidic devices are also of great interest [14]. Microfluidic devices enable the characterization and manipulation of fluids, leading to the development of important applications that impact multiple engineering and scientific research fields [15]. However, because sub-meter waves offer a wide range of benefits to the chemical and biological fields, researchers have developed diverse strategies to build integrated microfluidic systems [16]. Many researchers are using microfluidic devices for various biochemical applications [17]. Thus, the integration of microelectronics and microfluidics can greatly expand the capabilities of self-contained lab-on-a-chip technologies. Electronic components can be integrated into microfluidic systems using a variety of techniques [18].

In particular, microfluidics can also be applied in biotechnology. Three-dimensional bioprinting, a technique in which cell-laden biomaterials are stacked in layers to create cellular tissue constructs, is currently being developed. Three-dimensional bioprinting includes the development of cell-laden bioinks [19]. Microgels have been used as hydrogels for cellular tissue repair [19]. Sanskrita et al. prepared dispersed microgels for cell encapsulation and 3D cell culture. They devised a new method to fabricate microgels based on a microfluidic system and formed cell-laden constructs, namely, intrafibrous silk–gelatin hydrogels (microgels) [20], by enzymatic crosslinking and ultrasonic physical crosslinking. However, photocrosslinking is commonly used in 3D bioprinting because it provides better control. Therefore, mixing silk fibrolin and gelatin by photocrosslinking is an attractive strategy for 3D construction [20].

Enzymes are biological molecules that greatly accelerate the rate of chemical reactions in living systems. The measurement of enzyme kinetics improves our understanding of many metabolic processes in cellular systems and facilitates the optimization of enzymes for biosensing and industrial applications [21]. Microfluidics is a powerful and promising platform for improving enzyme kinetics measurements due to its unique ability to control small amounts of fluids and solids with high time resolution [22]. When combining enzymes with microfluidics, several aspects need to be considered, such as the integration of enzymes into the system. This can be achieved by immobilizing biomolecules on supporting surfaces such as electrodes, microfluidic channels, paper, microtubes, or microparticles, or by injecting them as a solution into the system at the time of measurement [23]. Injectable granular gels made of microgels have recently shown great potential for tissue regeneration applications, allowing administration through minimally invasive surgery, electron transfer, and highly efficient exchange of nutrients/waste products.

#### 2.2.4. Using Polymers, Photoimine Crosslinking, or Gels

Using a microfluidic droplet-based technique, Chuanfeng An et al. [24] demonstrated that the introduction of a photoinduced imine crosslinking (PIC) polymerization route for microgel formation can lead to the fabrication of granular gels via bottom-up assembly of cell-containing microgels fabricated by PIC [24]. Therefore, they decided to synthesize imine-crosslinked chitosan using PIC.

PIC chemistry reduces oxygen inhibition in free radical polymerization, promoting improved fabrication precision, accelerated gelation rate, and enhanced network strength. It may thus be a powerful tool for the design and application of next-generation tissue engineering strategies. Photoinduced imine crosslinking chemistry has been introduced into microgel crosslinking and demonstrated to enable the fabrication of microgels with improved matrix uniformity, accelerated gelation process, and improved mechanical strength. However, conventional injectable hydrogels generally induce chemical reactions to enable gelation, which results in reduced cell viability and the formation of homogeneous hydrogel networks due to rough and uneven crosslinking [24].

Recent advances in microgel fabrication using microfluidic droplets have demonstrated that this technique is effective and versatile for cell encapsulation in terms of high loading efficiency, high throughput, and improved controllability of microgel properties.

## 3. Detailed Aspects of Biofuel Cells Used in Microfluidics

### 3.1. Types of Biofuel Cells and Basic Principles

**Figure 3:**
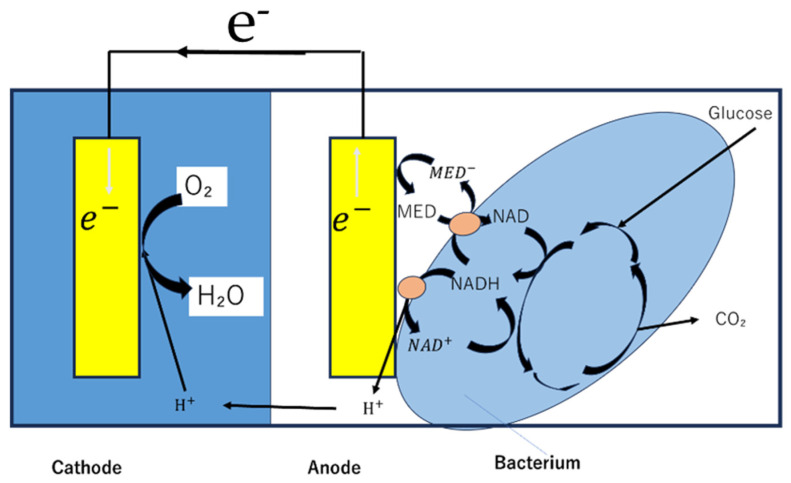
A typical two-compartment layout of a microbial biofuel cell. Adapted from Ref. [6].

**Figure 4:**
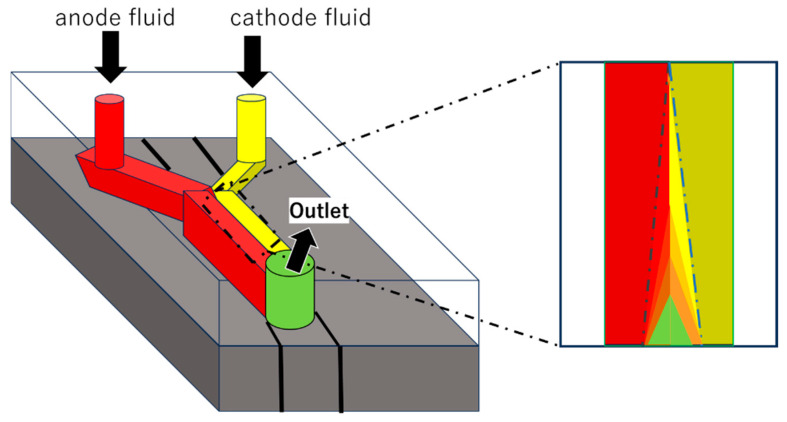
Schematic of a membraneless fuel cell with microfluidic channels. Adapted from Ref. [6].

An enzymatic biofuel cell uses natural enzymes as catalysts to oxidize fuels (glucose, lactate, ethanol, etc.) and convert chemical energy into electrical energy. At the anode, oxidizing enzymes such as glucose oxidase and glucose dehydrase oxidize the fuel (Figure 3), while at the cathode, reductive enzymes such as laccase and bilirubin oxidase reduce oxygen [3,25]. For microbial fuel cells, microorganisms (such as yeast or bacteria) are used as catalysts. Microorganisms have the advantage of being longer-lived than enzymes and being able to decompose more complex organic matter. On the other hand, their power density is generally several orders of magnitude lower than that of enzymatic fuel cells [3,5,7]. Microfluidic technology can suppress the mixing of reactants without a physical separation membrane by having the anode fluid and cathode fluid flow in parallel. This method eliminates the problems of membrane manufacturing costs and degradation, and simplifies the overall cell structure (Figure 4) [2,3,6].

### 3.2. Electrode Materials and Nanotechnology Applications

Carbon nanotubes (CNTs) have been widely used to improve enzyme processing and electron transfer. Electrodes consisting of compressed CNTs and enzymes have realized high-power glucose biofuel cells [2,3,25]. Single-walled CNTs (SWCNTs) modified with DNA were used to immobilize Gox and improve the electrical properties of enzyme biofuel cells (EBFCs). Graphene and reduced graphene oxide (rGO) have also been used as enzyme immobilization supports due to their high electrical conductivity and surface area [6,25].

**Figure 5:**
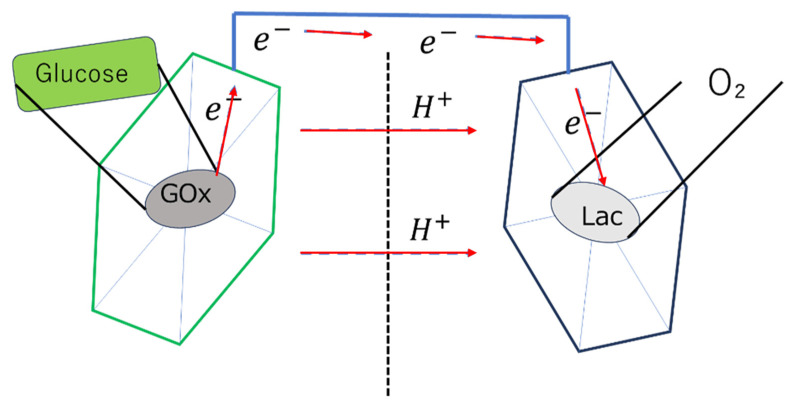
Schematic representation of GOx bioanode and laccase (lac) biocathode for glucose/oxygen biofuel cell. Anode (Left): Immobilized glucose oxidase (GOx) enzyme oxidizes glucose and releases electrons (e^−^). Cathode (Right): Immobilized laccase enzyme reduces the oxidizing agent (O_2_) to produce water. This battery has an open-circuit voltage of 0.7 V and a maximum power density of 7.05 ± 0.05 mW cm^−2^. Additionally, after 60 days of cyclic charging and discharging, the capacity retention was maintained at 84.2%.

Micropillar electrodes made of PDMS increased the effective electrode surface area by 1.8 times compared to flat electrodes. This resulted in a 40.5-fold increase in the average power density in phosphate-buffer solution [9]. A microbial fuel cell using 3D porous graphene/nickel foam electrodes achieved a high power density of 661 W/m^3^ [7]. A spider web-like 3D substrate was designed to immobilize GOx and Lac(laccase) and showed an OCV of 0.7 V and a maximum power density of 7.05 ± 0.05 mW/cm^2^ (Figure 5) [25].

Gold nanoparticles (AuNPs) have been used to modify electrodes and are active in the electrooxidation of glucose. EBFC using microelectrodes modified with AuNPs has been successfully used to generate electricity in tears [3,25]. Ferritin (iron storage protein) has been applied as a bioanode material by combining SWCNTs with nanosized iron oxide technology [25].

When glucose is used as fuel, GOx or cellobiose dehydrogenase (CDH) is often used as the anode catalyst, and Saccharomyces cerevisiae (baker’s yeast) is often used as the microbial catalyst [3,8,9]. Laccase and bilirubin oxidase (BOD) are commonly used as cathode catalysts that reduce oxygen [3].

Enzymes are immobilized on the electrode surface by physical adsorption, crosslinking with glutaraldehyde, electrostatic interactions with polymers (e.g., polyethyleneimine), and covalent bonding [3,5,26]. Cosnier et al. developed a simple method to mechanically compress CNTs and enzymes to fabricate disk-shaped electrodes, and reported good performance [27].

### 3.3. Direct or Mediated Electron Transfer

Direct electron transfer mechanism allows electrons to be transferred directly between the enzyme and the electrode without the aid of a mediator, which is essential for simplifying cell design. Research by Dutton et al. has shown that for efficient electron tunneling to occur, the distance between the redox centers needs to be less than 14 Å [3,4].

On the other hand, mediated electron transfer (MET) employs redox mediators to shuttle electrons between the enzyme and electrode, facilitating communication when direct contact is hindered [3,9].

### 3.4. Operations Using Fuels in Biological Solutions

A PDMS µMFC using S. cerevisiae operated in human plasma containing 4.2 mM glucose recorded an OCV of up to 488.1 mV, a current density of 30.2 µA/cm^2^, and a power density of 401.2 nW/cm^2^ [9]. Although the glucose concentration in tears is very low compared to blood (e.g., 13 µM), it can be used as a fuel source for EBFCs [3]. In a study by Falk et al., a CDH/ bilirubin oxidase (BOx)-immobilized AuNP-modified microelectrode was operated in human tear fluid, achieving an OCV of 0.57 V and a maximum power density of 3.5 µW/cm^2^ [3]. A wearable EBFC fueled by lactate has been developed that delivered 5–70 µW/cm^2^ of power from the exercising human body [25]. A wearable EBFC fueled by ethanol demonstrated a maximum output of 0.65 µW/cm^2^ from the forehead and 1.01 µW/cm^2^ from the forearm in subjects after drinking alcohol [25]. In a study by Falk et al., a CDH/BOx-based EBFC was operated in human saliva, achieving power densities up to 2.1 µW/cm^2^, but with lower performance than in blood [3].

### 3.5. Evaluation of Performance of Cells

Regarding performance evaluation, various indicators and figures have been reported in previous papers. Their performance is shown in Table 2 below. For specific numerical data, please refer to the original papers. Microbial cells demonstrated power densities of 2.3 nW/cm^2^ for silicon-based µMFCs and 401.2 nW/cm^2^ (in human plasma) for PDMS µMFCs [8,9]. Enzymatic cells tend to have power densities several orders of magnitude higher than microbial cells: a glucose EBFC with a Y-shaped microchannel has a power density of 0.11 mW/cm^2^, a glucose EBFC with compressed CNT electrodes has a power density of 1.3 mW/cm^2^, and a methanol fuel cell with a Pt-Ru anode and laccase cathode has a power density as high as 8.5 mW/cm^2^ [5,25]. Open-circuit-voltage (OCV) µMFC using S. cerevisiae showed a maximum of 475.3 mV in phosphate buffer and 488.1 mV in human plasma. Falk et al. [3]. developed an EBFC operating in tear fluid that recorded an OCV of 0.57 V. The enzyme’s lifetime is a major challenge for BFCs, and is on the order of hours to days. Their EBFC had a half-life of 30 h in buffer, 8 h in plasma, and 2 h in blood [3,5,25]. Microorganisms have a much longer lifespan than enzymes because they reproduce autonomously [5]. Furthermore, Siu and Chiao et al. reported that the Coulombic efficiency of a PDMS µMFC reached 9.0% to 14.7% after 60 min of operation in human plasma [9].

### 3.6. Towards Implantable and Wearable Devices

**Figure 6:**
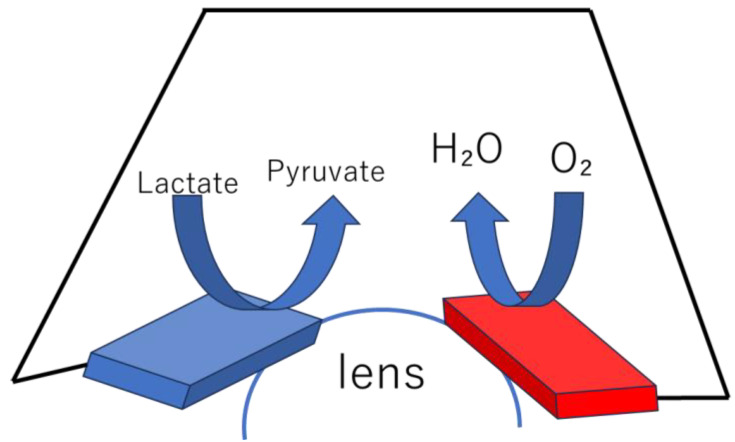
EBFC applied to contact lenses.

**Figure 7:**
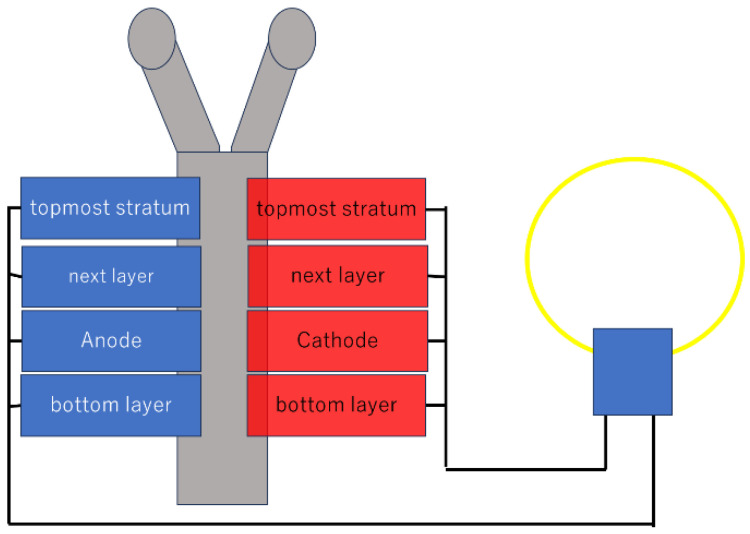
Paper-based EBFCs stacked on shelves (red: cathode; blue: anode).

There have been several reports on the performance of battery systems that utilize the properties of biomaterials for attractive applications. Cinquin et al. implanted glucose-fueled EBFCs into the abdominal cavity of rats and showed that they maintained a stable voltage (0.25 V) after 40 days [25]. Katz et al. implanted EBFCs into live snails and crayfish. In particular, in the crayfish, they achieved the voltage (2.8 V) and power (90 µW) required to drive a pacemaker by connecting five cells in series [25]. Lee et al. implanted an EBFC and brain stimulator into pigeons and successfully generated 0.08 mW of power and wirelessly transmitted light in vivo using only glucose and oxygen in the body [25]. Magner et al. developed an EBFC applicable to contact lenses using nanoporous gold (NPG). This battery is made by placing enzyme-modified NPG electrodes between two commercially available contact lenses. Lactate oxidase (LOx) is immobilized on the anode, and bilirubin oxidase (BOD) is immobilized on the cathode. When tested in artificial tears at equilibrium with air, this EBFC exhibited an open-circuit voltage of 380 ± 28 mV and a maximum power density of 1.7 ± 0.1 µW/cm^2^. This demonstrates the potential for utilizing tears as a renewable energy source and the possibility of applying this technology to power supply for micro-wearable devices such as contact lenses. A power density of 1.7 ± 0.1 µW/cm^2^ was achieved in simulated tear fluid (Figure 6) [25]. Goel et al. created a paper electrode based on multi-walled carbon nanotubes (MWCNTs) and fixed tetrathiofurane (TTF), an electron transfer mediator, and the enzyme GOx (glucose oxidase) to the anode by crosslinking them with glutaraldehyde. The cathode was immobilized with the enzyme laccase. A single-chamber EBFC was constructed by connecting four Y-shaped paper microchannels in series. These achieved a stable open-circuit potential of up to 1.65 V and a maximum power density of 46.4 μW/cm^2^ at 0.8 V. This output power is sufficient to supply power to small portable devices. The Y-shaped channels demonstrated the ability to sustainably and stably supply microfluidic fuel and oxidant, thereby enabling stable power output (Figure 7) [25]. Miyake et al. developed a bracelet-type EBFC that uses lactic acid as fuel and succeeded in powering an electronic watch by connecting six cells in series [25].

### 3.7. Issues to Be Overcome Regarding Battery Materials

Enzymes are easily denatured or inactivated by the effects of temperature, pH, and chemicals. In addition, the lifespan of an enzyme is generally about 10 days, which significantly limits its application to devices that require continuous operation for several months to several years.

As examples of relatively high-power EBFCs, a glucose fuel cell using compressed CNT electrodes with a power density of 1.3 mW/cm^2^ and a methanol fuel cell combining a Pt-Ru anode and a laccase cathode with a power density of 8.5 mW/cm^2^ have been reported. However, these values are not as high as those achieved by conventional fuel cells. This limited power density is a factor that limits the range of applications of biofuel cells.

Upon contact with biological fluids, proteins adsorb onto the surfaces of electrodes and membranes, leading to inhibition of mass transport. The biological response after implantation physically blocks the supply of fuel to the device. Some biological fluids, such as tears, have lower glucose concentrations compared to blood, directly limiting their performance. These interactions with the biological environment are also obstacles that must be overcome to maintain the long-term stability of implantable devices.

To build a practical power supply system, it is necessary to develop manufacturing techniques that ensure uniformity in cell performance, strategies to avoid problems at the system level, and a self-operating design that does not require external power.

### 3.8. Recent Research Related to Some Aspects

The topic covered in this review is cutting-edge and has few research examples essentially. Recent research exists for each keyword, but comprehensive studies covering all aspects are scarce as follows.

Carbon-Based Nanomaterials: CNTs and rGO serve as excellent electrode platforms for BFCs due to their high conductivity and biocompatibility [26]. CNT-modified electrodes have been reported to significantly enhance photocurrent density and improve the performance of thylakoid membrane-based photosynthetic biofuel cells [26]. 3D structures based on rGO have been shown to improve photocurrent density by providing high electrode surface area, electron transport pathways, and enabling close contact with thylakoid membranes [26]. Metal Oxides and Conductive Polymers: Metal oxides and conductive polymers are also incorporated into nanostructured electrodes. Conductive polymers, with their excellent flexibility and biocompatibility, have been shown to be ideal materials for printable and wearable thylakoid membrane-based photosynthetic biofuel cells [26].

Flexible and printable electrodes: Research is also advancing towards realizing scalable, lightweight, and disposable BFC platforms [26]. A method using inkjet printing technology to directly deposit CNT electrodes and thylakoid membranes onto paper substrates has reported performance exceeding algae-based platforms by more than 20 times [26].

Evolution of Nanomaterials and Electrodes: Nanomaterials are considered essential for dramatically enhancing BFC performance by improving electrode surface area and conductivity [27].

Improved Electron Transfer Mechanisms: Redox mediators play a central role in facilitating charge transfer between the photosynthetic system and the electrodes [28]. Synthetic mediators such as quinones and phenazinemethanesulfonate (PMS) have been shown to function as more efficient electron shuttles than natural mediators [28]. PMS, in particular, has been reported to mediate highly efficient charge extraction from thylakoids, generating a photocurrent approximately 1000 times higher than that produced by p-benzoquinone (PBQ) [27,28].

Wearable Devices using Photovoltaic Cells: Converting sunlight and indoor light into electricity. Organic solar cells and perovskite solar cells, made from lightweight, flexible materials with high efficiency, are expected to power wearable sensors when attached to clothing [29,30]. Perovskite solar cell modules exceeding 31% efficiency under indoor light have also been reported [29,30].

Biofuel cells: Generate electricity using chemical energy from bodily fluids like sweat or blood. They are highly biocompatible and use enzymes as catalysts, demonstrating operation under mild conditions like body temperature and neutral pH [30,31]. They are also considered to pose almost no explosion risk. A bracelet-type device incorporating flexible, fiber-like BFCs has been developed, potentially enabling high-power generation [30].

Tritanic generators: These generate electricity by repeatedly contacting and separating materials to utilize the triboelectric effect. With a simple structure, it has been demonstrated to generate high-power electricity from human movements (sliding, bending, stretching, tapping, etc.). Textile tribogenerators woven with conductive fibers or nylon threads can be integrated into clothing and have been shown to enable monitoring of breathing and pulse [29,30]. Tribogenerators based on transparent, stretchable hydrogels have also been developed.

Piezoelectric Generators: Utilize the piezoelectric effect to convert mechanical stress (stretching, bending, etc.) into electrical energy. Piezoelectric generators feature high voltage, high power density, and a wide frequency response range, making them promising power sources for wearable electronics and medical devices. Polymer materials such as polyvinylidene fluoride are widely reported to be used. They are incorporated into shoe soles to generate power from walking and also serve as sensors detecting finger movements [29,30].

Thermoelectric generators: Utilize the Seebeck effect to convert the temperature difference between body heat and the surrounding environment into electricity. They provide a stable power source as they are less dependent on external environments or motion. Development of materials that achieve both low thermal conductivity and high-power is reported as crucial. A wristband incorporating a high-output, flexible material has been developed, with examples shown of it powering a multi-sensor bracelet that continuously monitors temperature, humidity, and activity levels [29,30].

Microfluidics Changes in Materials Themselves: Materials for microfluidic devices are reported to have evolved over time. Initially, inorganic materials like silicon and glass were primarily used, but these are costly, and silicon is opaque, making it unsuitable for optical detection. Consequently, the focus shifted to more affordable and versatile polymer materials [32,33].

PDMS has become particularly widespread. PDMS offers numerous advantages, including transparency, chemical inertness, biocompatibility, flexibility, and gas permeability. However, its hydrophobic surface also has the drawback of causing non-specific molecular adsorption. Recently, inexpensive and easily processable paper materials have also gained attention [32,33,34].

Applications have been demonstrated particularly in portable diagnostic devices and wearable technology [32].

Advances in Manufacturing Methods: Manufacturing methods for microfluidic devices are also diversifying. Traditional techniques include hot embossing, injection molding, and soft lithography. However, recent reports indicate that 3D bioprinting is emerging as a promising method [33,35].

Soft lithography is a low-cost technique widely used for rapid prototyping of PDMS devices. However, PDMS molds are prone to elastic deformation, potentially causing channel size variations [32,34].

Light-based methods utilize photopolymerization (light polymerization) or photodecomposition to create complex-shaped microfluidic channels. They offer advantages such as high resolution and multi-material capability but are reported to require expensive equipment and complex processes [32].

3D printing has gained attention due to its advantages of design flexibility, rapid prototyping, and low cost. Specifically, 3D bioprinting enables the fabrication of complex 3D structures incorporating living cells and is utilized for creating organ-on-a-chip devices [32].

## 4. Conclusions and Perspectives

In this review, we investigated electrode materials, biocatalyst immobilization techniques, electron transfer mechanisms, and the possibility of application to microfluidic devices to improve the performance of biofuel cells. Compared to conventional fuel cells, EBFCs operate under mild conditions and have high biocompatibility and specificity, but issues remain in terms of power density, stability, and enzyme lifespan. Regarding biocatalysts and immobilization techniques, GOx and CDH are widely used as anode catalysts for glucose fuel, and laccase and BOD are widely used as cathode catalysts. Various methods such as physical adsorption, crosslinking, electrostatic interactions with polymers, and covalent bonding are used to immobilize these enzymes, which contribute to maintaining enzyme activity and improving electron transfer efficiency.

New challenges include stabilizing the catalyst and increasing the device life, improving the output density, overcoming performance degradation due to interactions with the biological environment, and establishing system integration and control. In addition, advanced electrode designs using nanomaterials such as carbon nanotubes, graphene, and 3D porous structures have greatly contributed to improving output performance by increasing the effective surface area and improving the enzyme immobilization efficiency.

In the future, important challenges for the practical application of biofuel cells include extending the enzyme life, further improving the electron transfer efficiency, preventing the leakage of mediators and enzymes, and ensuring resistance to interactions with the biological environment. The progress of microfluidic biofuel cells that use biological fluids as a fuel source will not only contribute to the development of sustainable and environmentally friendly next-generation energy devices but also provide great prospects for future medical and wearable applications.

Enzymes (including laccase) are easily deactivated by external factors such as temperature, pH, metal ions, and oxidizing species derived from the substrate, and their long-term stability is one of the important issues for practical use of biofuel cells. In a future study, we will aim to stabilize and protect the active center of laccase by complexing it with a chiral salen-type Fe(II) complex and a chitosan-Schiff base Fe(II) complex, thereby extending the life of the enzyme function.

## Figures and Tables

**Table 1:** Power density, stability, and lifespan of fuel cells. Reprinted from Ref. [6].

Fuel Cell Types	Fuel	Oxidant	Cell Voltage (V)	Maximum Current Density (mA/cm^2^)	Maximum Power Density (μW/cm^2^)
Microbial	Glucose	Air	0.52	-	0.0494
Enzymatic	Methanol	Air	0.65	50	8.5
Enzymatic	Glucose	Oxygen	0.12	0.55	0.021
Microfluidic/ microbial	Glucose	[Fe(CN)_6_]^3−^	0.45	0.016	2.3 × 10^−6^
Microfluidic/ microbial	Glucose	[Fe(CN)_6_]^3−^	0.488	0.03	4.01 × 10^−4^
Microfluidic/ microbial	Lactate	[Fe(CN)_6_]^3−^	-	0.013	1.5 × 10^−4^
Microfluidic/ microbial	Acetate	Oxygen	0.619	0.14	0.012
Microfluidic/ microbial	Ethanol	Air	0.34	0.053	5.0 × 10^−3^
Microfluidic/ microbial	Acetate	Air	0.4	0.45	25 × 10^−3^
Microfluidic/ microbial	Glucose	Oxygen	0.55	0.065	-
Microfluidic/ microbial	Glucose	Oxygen	0.55	0.69	0.11

**Table 2:** Comparison of key performance indicators (OCV, power density, and stability).

Battery Type	Type of Catalysts	OCV (V)	Power Density (μW/cm^2^)	Stability
EBFC	GDH, Dp, VK_3_	0.8	530	NA
EBFC	GDH, Dp, VK_3_	0.55	32	>18 h
Microbial BFC	ADH, PMG	0.34	53 ± 9.1	NA
Microbial BFC	S, cerevisiae, MB	0.488	401.2	>60 m
EBFC	S, cerevisiae, MB	0.475	364.1	>60 m
EBFC	ADH	0.61–0.82	1000–2040	>450 days
Microbial BFC	S, oneidensis MR-1	0.6	1.5	160 h
Microbial BFC	S, cerevisiae, MB	0.3–0.5	2.3	40 m

## Data Availability

No new data were created or analyzed in this study. Data sharing is not applicable to this article.

## Abbreviations

The following abbreviations are used in this manuscript:

PEM	proton exchange membrane
MEMS	microelectromechanical systems
SAV	surface area-to-volume
BOD	bilirubin oxidase
GDH	glucose dehydrogenase
GOD	glucose oxidase
ADH	alcohol dehydrogenase
OKABTS	azino-bis(3-ethylbenzothiazoline-6-sulfonic acid
CLEA	cross-linked enzyme aggregates
PIC	photoinduced imine crosslinking
FRP	free radical polymerization
PEGDA	polyethylene glycol diacrylate
GelMA	methacrylated gelatin
MED	Mediator
CNT	Carbon nanotube
SWCNT	Single-walled Carbon nanotube
DNA	deoxyribonucleic acid
Rgo	reduced graphene oxide
OCV	Open Circuit Voltage
AuNP	Gold nanoparticle
CDH	cellobiose dehydrogenase
NPG	nanoporous gold
